# The Impact of Integrated Infant and Young Child Feeding and Micronutrient Powder Intervention on Feeding Practices and Anemia in Children Aged 6–23 Months in Madagascar

**DOI:** 10.3390/nu9060581

**Published:** 2017-06-07

**Authors:** Lindsey M. Locks, Ietje Reerink, Amal Tucker Brown, Smaila Gnegne, Noelimanjaka Ramalanjaona, Simeon Nanama, Christopher P. Duggan, Aashima Garg

**Affiliations:** 1 Harvard TH Chan School of Public Health, 677 Huntington Ave, Boston, MA 02115, USA; lml395@mail.harvard.edu (L.M.L.); Christopher.Duggan@childrens.harvard.edu (C.P.D.); 2 United Nations Children’s Fund (UNICEF) Headquarters, 3 United Nations Plaza, New York, NY 10017, USA; 3 Population Services Internationl (PSI), Fiaro, Antananarivo BP 7748, Madagascar; ietjereerink@yahoo.com (I.R.); noelimanjakar@psi.mg (N.R.); 4 United Nations Children’s Fund (UNICEF), Maison Commune des Nations Unies Zone Galaxy Andraharo, Antananarivo BP 732, Madagascar; atuckerbrown@unicef.org (A.T.B.); sgnegne@unicef.org (S.G.); snanama@unicef.org (S.N.); 5 Boston Children’s Hospital, 333 Longwood Avenue, Boston, MA 02115, USA

**Keywords:** micronutrient powders (MNP), Infant & Young Child Feeding (IYCF), children, anemia, community health workers

## Abstract

This study assesses the impact of an integrated infant and young child feeding (IYCF) and micronutrient powder (MNP) intervention on children’s risk of anemia and IYCF practices in Madagascar. Quantitative baseline and endline surveys were conducted in representative households with children 6–23 months from two districts, where an 18-month IYCF-MNP intervention was implemented. Relative risks comparing children’s risk of anemia and maternal IYCF knowledge and practices at baseline versus endline, and also at endline among MNP-users versus non-users were estimated using log-binomial regression models. 372 and 475 children aged 6–23 months were assessed at baseline and endline respectively. Prevalence of anemia fell from 75.3% to 64.9% from baseline to endline (*p* = 0.002); the reduction in the risk of anemia remained significant in models adjusting for sociodemographic characteristics (ARR (95% CI): 0.86 (0.78, 0.95), *p* = 0.003). In endline assessments, 229 out of 474 (48.3%) of children had consumed MNPs. MNP-users had a lower risk of anemia (ARR (95% CI): 0.86 (0.74, 0.99), *p* = 0.04) than non-users, after controlling for child’s dietary diversity and morbidity, maternal counseling by community-health-workers, and sociodemographic characteristics. Mothers interviewed at endline also had greater nutrition knowledge and were more likely to feed their children ≥4 food groups (ARR (95% CI): 2.92 (2.24, 3.80), *p* < 0.001), and the minimum acceptable diet (ARR (95% CI): 2.88 (2.17, 3.82), *p* < 0.001) than mothers interviewed at baseline. Integration of MNP into IYCF interventions is a viable strategy for improving children’s consumption of micronutrients and reducing risk of anemia. The addition of MNP does not negatively impact, and may improve, IYCF practices.

## 1. Introduction

Globally, over two billion people are deficient in key vitamins and minerals, particularly vitamin A, iodine, iron and zinc [1]. Nutritional deficiencies during the critical period from conception to two years of age increase the risk of mortality, morbidity, and long-term developmental delays [2]. Multiple micronutrient deficiencies often cluster within the same individuals and communities, thus making interventions that target multiple deficiencies particularly attractive and efficient [3,4,5]. Micronutrient powders (MNPs) may be particularly effective because the single-dose micronutrient packets are light-weight, shelf-stable, tasteless and odorless, and can be mixed with a variety of semi-solid foods [6]. The efficacy of MNPs has been well-established as demonstrated by recent meta-analyses of trials in children under 2 years [7,8] which concluded that prolonged, regular use of MNPs can reduce the prevalence of anemia by more than 25% and iron deficiency by more than 50%.

Despite the fact that MNP programs reach millions of children each year [9], there are only a handful of studies that have assessed how to best implement programs using MNPs at scale [10,11,12,13,14,15,16]. Studies have documented the use of MNP in a variety of contexts including emergency settings [10], as part of rural food distribution programs [15], as part of integrated child health programs [11], and the sale of MNP through social marketing [17]. Increasingly, MNPs are being integrated into large-scale infant and young child feeding (IYCF) programs [18,19]; however there is limited evidence demonstrating the impact of integrated interventions on IYCF practices and ultimately on children’s nutritional status [19]. In this study, we assess the impact of an integrated IYCF-MNP program in two rural districts in Madagascar on anemia risk in children aged 6–23 months and IYCF practices. 

## 2. Materials and Methods

### 2.1. Program Description

Project Fortidom (fortification a domicile in French, or home fortification), was a pilot program implemented as a collaboration between the Madagascar Ministry of Health (MoH), the National Nutrition Office (ONN), the United Nations Children’s Fund (UNICEF) and Population Services International/Madagascar (PSI). The integrated IYCF-MNP program introduced a branded MNP (Zazatomady) into selected parts of Madagascar and aimed to reduce anemia among children aged 6–23 months and also to improve IYCF practices. In the two rural districts where Fortidom was implemented (Vavatenina and Fénérive-Est), community-health workers (CHWs) distributed MNP using a social marketing approach [20]. (A separate distribution model was piloted in urban Antananarivo and Fianarantso; however, quantitative data was not collected in the urban areas, and thus they are not included in the current study). A social marketing model was selected to motivate CHWs to sell MNP to mothers and to counsel them on how to integrate MNP into IYCF practices; the social marketing model was also intended to promote mothers’ investment in MNP to support consumption and adherence. As part of the program incentive structure, CHWs received 10 free boxes of MNP as a starter stock and were then able to buy subsidized MNP from community supply points for 100 ariary (~0.05 USD) per box. CHWs then sold boxes of 30 sachets of MNPs to mothers at a subsidized price of 200 ariary (~0.10 USD) and kept the profits or invested them in additional MNP. The subsidized prices—intended to be high enough to motivate CHWs, but low enough to remain affordable for low-income households—was determined by willingness-to-pay qualitative research prior to the implementation of the program.

Project Fortidom also provided CHWs with a 5-day training on IYCF and MNP using the UNICEF community-based IYCF training tools [20] that were adapted for the program context. ONN and MoH federal and regional staff conducted the trainings, with technical support from UNICEF and PSI staff. Some CHWs had already received MoH IYCF training; when this was the case, refresher IYCF trainings with the added MNP modules were condensed to 3 days. CHWs then incorporated MNP and IYCF counselling into their regular activities including their monthly growth monitoring sessions and their monthly mothers group meetings. Some CHWs also made home visits to women in their catchment areas who had recently given birth or who had missed several group meetings or other community outreach activities to check on the mothers and infants. In home visits, CHWs counselled mothers on breastfeeding and when age-appropriate, they also discussed food diversification and MNP use. During Fortidom, key messages on IYCF and MNP were re-iterated through radio messages (MNP radio messages discussed the long-term benefits of MNP, the appropriate use of MNP, and the price and locations for purchase; while IYCF radio messages focused on the value of breastfeeding and dietary diversity for children over 6 months). Fortidom also introduced CHW job aids including a flip chart and an interactive wallchart that allowed mothers to discuss which foods they fed their children based on pictures of locally available foods. 

### 2.2. Study Design and Data Collection

Fortidom began distributing MNP in February 2013; at that time the two rural districts had a combined population of 610,759 inhabitants including 48,860 pregnant and lactating women, and 33,775 infants and children 6 to 23 months [21]. Quantitative household surveys were conducted prior to program initiation in October-November 2012 and after implementation in April 2014. PSI Madagascar and MOH staff conducted 5-day trainings for all study interviewers (MDs or paramedical staff). The training included information on study methodology, the survey questionnaires, and ethical considerations. For haemoglobin assessment, data collectors were trained to prick children’s fingers to obtain capillary blood, and to use the child’s heel if they had difficulty obtaining blood from the child’s finger. They were instructed to wipe away the first two drops of blood using a clean, dry gauze, and to collect the third drop of blood and directly into a microtainer for analysis. All hemoglobin was assessed using HemoCue^®^ Hb 301 (Hemocue, Ängelholm, Sweden). 30 interviewers (six supervisors and 24 interviewers) were selected to collect data at baseline and endline. The same trainers and training tools were used at baseline and endline (with the addition of a new MNP and program exposure section at endline), and approximately one-third of interviewers participated in both baseline and endline surveys. Two PSI Madagascar quantitative researchers and three MHO experts coordinated and supervised the fieldwork.

Stratified multi-staged sampling was used to collect representative data from households with children age 6–23 months in the two districts. Lists of 47 and 97 fokontany (municipalities) in Vavatenina and Fénérive-Est respectively, were used to select 18 fokontany in each district using probability proportional to size. In each sampled fokontany, a census was used to randomly select households. Sample size calculations designed to detect a 10% difference in the prevalence of anemia with an alpha level of 0.05 and an assumed proportion of MNP use of 50%, indicated that 858 infants aged of 6–23 months of age would be needed for each survey. Based on Demographic and Health Survey data indicating approximately one-quarter of households would have a children between the age of 6–23 months, survey interviewers visited 3240 and 4085 households at baseline and endline. Households that were unavailable for interviews were not replaced. At each household, the household head was first interviewed to obtain a list of all household members. If the household included any children 0–23 months, the household head and caregiver were administered follow-up questionnaires. Household heads were asked socioeconomic and demographic questions, then caregivers of children 0–23 months were interviewed about nutrition and IYCF (endline and baseline) and program exposure including questions on knowledge and practices relating to MNP (endline only). Caregivers were also asked to read headlines from a local newspaper to assess literacy. Caregivers of infants aged 6–23 months were asked their consent for their child to participate in anemia testing; these caregivers were also asked additional questions on complementary feeding. Given that the outcomes of interest in this manuscript focus on anemia, complementary feeding, and MNP-use, all analyses are limited to children aged 6–23 months.

### 2.3. Ethical Approval

Ethical approval for this study was provided by the Madagascar Research Ethics Committee, and all caregivers provided written informed consent to participate in the study. 

### 2.4. Outcome Variables

Children’s hemoglobin was assessed using HemoCue^®^ Hb 301 (Ängelholm, Sweden). Anemia was defined as hemoglobin <110 g/L; given that all intervention areas were at altitude levels less than 1000 m above sea-level, altitude adjustment was not necessary. IYCF knowledge and practices were assessed using a questionnaire adapted from the WHO/UNICEF document on ‘Indicators of Infant and Young Child Feeding’ [22]. Mothers were asked whether they were still breastfeeding, and if so, how many times they had breastfed their child in the previous day. A modified 24-h recall using prompt cards with photos of common local foods was also used to help the respondents recall meals and snacks given to their child. Mothers reported all foods the child consumed in the previous day and the frequency of feeding, and data collectors coded responses based on standard food groups. The endline questionnaire organized responses into the seven food groups recommended by WHO/UNICEF: (1) grains/roots/tubers; (2) legumes/nuts; (3) dairy; (4) flesh foods; (5) eggs; (6) vitamin-A-rich fruits/vegetables; and (7) other fruits/vegetables. The baseline questionnaire organized responses by 11 food groups that were collapsed during data analysis to the same seven groups. In accordance with the WHO/IYCF indicators: minimum dietary diversity was defined as ≥4 of seven food groups; minimum meal frequency as solid/semi-solid foods ≥2×/day for breastfed infants aged 6–8 months, ≥3×/day for breastfed children 9–23 months, and ≥4×/day for non-breastfed children 6–23 months. Minimum acceptable diet was defined as minimum dietary diversity and minimum meal frequency for children who were breastfed; for children who were not breastfed, it was defined as minimum meal frequency, at least four out of 6 food groups (based on the 7 food groups above excluding dairy), and at least two milk feedings per day. At endline, mothers were also asked to report whether they had ever fed their child MNP, and if so, how many sachets they had used.

### 2.5. Data Analysis

Means with standard deviations and frequencies were used to describe sample characteristics at baseline and endline. Relative risks for anemia and maternal IYCF knowledge and practices comparing endline to baseline samples were estimated using log-binomial regression models [23,24]. When the binomial model did not converge, the poisson distribution was used [25]. The differences in means for continuous variables were obtained from linear regression models. Covariates for multivariate models were selected a priori based on a review of the literature and include: child’s sex and age; district; urban vs. versus rural (within pilot districts); household asset score (0, 1 or ≥2 from 3-item list of transportation (bicycle/car), telephone (cell/landline) and electronics (radio/television)); household head's occupation; and maternal literacy. Models were first built for each district separately; however, since there were no substantial differences, the two districts were collapsed and population prevalences and means at baseline and endline were weighted based on the size of district population, as were the models for the relative risks and differences in means.

Endline data was also analyzed to assess the relationship of MNP consumption with children’s anemia risk and IYCF practices with log-binomial and linear regression models using the same covariates described above. Since MNP consumption could be an effect modifier of the relationship between CHW exposure and anemia or IYCF, we first used likelihood ratio tests to compare models with CHW exposure, MNP consumption, an interaction term between CHW exposure and MNP consumption, and demographic covariates, to reduced models that did not have the CHW-MNP-use interaction term. When this was not significant, we removed the interaction term and modelled MNP consumption as our predictor of interest. For models for IYCF practices, we considered MNP consumption a proxy for program exposure—since CHWs sold MNP as part of an integrated intervention that included counselling on nutrition and IYCF–and thus multivariate models for IYCF practices adjust for demographic characteristics only. For hemoglobin and anemia, we considered the added micronutrients in MNPs as the mechanism of interest, and thus models for anemia and hemoglobin are further adjusted for maternal CHW-exposure, child’s dietary diversity score and child’s morbidity in the previous 2 weeks.

We also identified predictors of whether mothers ever fed their children MNP and whether the mother ever fed the child ≥30 sachets of MNP, by first conducting univariate log-binomial regression models with all variables of interest, and then retaining all variables that were significant at the *p* < 0.2 level in multivariate models. Because attending a CHW meeting and hearing radio messages were co-linear with several maternal knowledge and perception questions, we first built multivariate models without the knowledge/perception indicators and reported these results for all variables. We then built separate multivariate models with maternal knowledge and perception indicators to obtain multivariate estimates for these indicators. All analyses were conducted in SAS 9.4 (SAS, Cary, NC, USA).

## 3. Results

Interviewers identified 496 and 627 households with children aged 0–23 months at baseline and endline respectively, of whom 95.0% and 85.8% completed the survey. The baseline and endline surveys included 372 and 475 children aged 6–23 months, 87.9% and 91.1% had their hemoglobin level assessed respectively. About one-third of households in our sample were located in an urban area that was part of the district capital in both the baseline and endline surveys, and the majority of household head’s cited agriculture or livestock as their primary source of income (Table 1). 

Over 95% of children in both the baseline and endline surveys were fed by their mothers (hereinafter, we refer to all caregivers as mothers). In the endline survey, 391 (82.5%) of mothers reported that they had heard of Zazatomady, 229 (48.3%) reported they had fed their child at least one sachet of MNP, and 127 (26.8%) reported feeding their children at least 30 sachets (one box). Among mothers who fed their child MNP, the median number of sachets was 30 (interquartile range: 7, 60). The most frequently cited reason that mothers reported not trying MNPs was a lack of trust/understanding of MNP (36.6%). Among mothers who knew the correct price of Zazatomady (200 ariary), 93.7% reported that they considered the product affordable. Among mothers who had tried MNP but stopped using it, 46.4% of mothers indicated they stopped because their child refused to eat MNP.

Children in the endline survey had a significantly reduced risk of anemia compared to children in the baseline survey (ARR (95% CI): 0.86 (0.78, 0.95), *p* = 0.003) (Table 2). Mothers in the endline survey were also significantly more likely to have heard of iron deficiency (ARR (95% CI): 2.41 (1.83, 3.18), *p* < 0.001), be able to identify a consequence of anemia (ARR (95% CI): 5.48 (4.14, 7.25), *p* < 0.001), and identify children 0–23 months as particularly vulnerable to anemia (ARR (95% CI): 8.48 (5.04, 14.26), *p* < 0.001). They were also significantly more likely to have breastfed their child (ARR (95% CI): 1.07 (1.01, 1.14), *p* = 0.02) and provide the minimum dietary diversity (ARR (95% CI): 2.92 (2.24, 3.80), *p* < 0.001) the previous day than mothers in the baseline survey. In particular, mothers in the endline survey were significantly more likely to feed their children dairy, vitamin-A-rich fruits/vegetables and other fruits/vegetables. It is worth noting, however, that the mean number of food groups remained below the recommended four at both baseline and endline (mean ± SD: 2.35 ± 1.10 and 3.35 ± 1.31 respectively). Children in the endline survey were also more likely to receive the minimum acceptable diet (ARR (95% CI): 2.88 (2.17, 3.82), *p* < 0.001), though feeding frequency was not significantly different in multivariate models. There was also a significant increase in the proportion of sick children fed more breastmilk and more solid foods during illness. 

In multivariate models adjusting for sociodemographic characteristics, we found that mothers who fed their children MNP were significantly more likely to feed their children solid foods (ARR (95% CI): 1.03 (1.00, 1.07), *p* = 0.046 for children 6–23 months) and the minimum dietary diversity (ARR (95% CI): 1.22 (1.01, 1.47), *p* = 0.04) than mothers who did not feed their children MNP (Table 3). In particular, MNP users were significantly more likely to feed their children dairy and vitamin A-rich fruits/vegetables than non-users. In models for hemoglobin and anemia risk, that further adjusted for maternal CHW exposure, child’s dietary diversity score and child morbidity, MNP users had a significantly lower risk of anemia (ARR (95% CI): 0.86 (0.74, 0.99), *p* = 0.04) and higher mean hemoglobin (Adjusted difference of means (95% CI): 4.5 g/L (1.9, 7.1), *p* < 0.001) than non-users. 

Significant predictors of MNP consumption in multivariate models were: living outside the district capital, child’s age, maternal literacy, maternal attendance at a CHW-IYCF meeting, and maternal exposure to radio messages on IYCF-MNP (Table 4). Mothers who had attended a CHW talk on IYCF were one and half times more likely to feed their children MNPs (ARR (95% CI): 1.55 (1.29, 1.85), *p* < 0.001), and there was a notable dose response relationship between frequency of radio message exposure and mothers feeding their children MNP, which culminated with mothers who heard the radio messages everyday being 2 times more likely than those who had never heard radio messages to feed their children MNP (ARR (95% CI): 1.97 (1.46, 2.65), *p* < 0.001). Notably older children were more likely to receive MNP: children who were 18–23 months were twice as likely to receive MNP than children aged 6–11 months (ARR (95% CI): 1.99 (1.57, 2.52), *p* > 0.001 and children aged 12–17 months were one and a half times more likely to consume MNP than the youngest children (ARR (95% CI): 1.51 (1.17, 1.94), *p* = 0.001). In models that included maternal perception variables, we found a significant association between feeding children MNP and maternal confidence in her ability to explain MNP benefits, identification of her CHW as the primarily source to obtain MNP, and her perception that other mothers in the community were using MNP for their children. 

Significant predictors of consumption of ≥30 sachets (one box) of MNP included child’s age, maternal CHW-exposure, district of residence (consumption of ≥30 sachets was more common in Fénérive-Este) and the amount each household spent on food per person per day (the less a household spent on food per person per day, the more likely they were to feed their child ≥30 sachets of MNP) (Table 5). In models that included maternal knowledge and perception variables, we found a significant association between feeding her child at least 30 sachets and maternal confidence in her ability to explain the benefits of MNP (ARR (95% CI) 1.46 (1.13, 1.89)).

## 4. Discussion

In this study of representative households with children aged 6–23 months from the two rural districts in Madagascar where Project Fortidom, an integrated IYCF-MNP intervention, was implemented, we found that the risk of anemia among children aged 6–23 months was significantly lower at endline than baseline. In order to determine whether the decline in anemia could be attributed to Fortidom, we conducted additional analyses from endline surveys assessing the relationship between program exposure (using MNP-use as a proxy) and anemia risk, and found that children who consumed MNP during the intervention had a significantly lower risk of anemia than children who had not consumed MNP, even after controlling the child’s morbidity, dietary diversity, maternal CHW-exposure, and sociodemographic characteristics. We also found that mothers interviewed at endline had superior nutrition knowledge and were more likely to feed their children the minimum dietary diversity and minimum acceptable diet than mothers at baseline. The role of Fortidom in improving IYCF practices was further supported in analyses of endline data that showed that mothers with greater program exposure (using MNP-use as a proxy) were more likely to feed their children solid food and a wider variety of food groups in the previous day than mothers with low program exposure.

Our findings not only show that CHWs are a viable platform for the distribution of MNP, but that utilizing CHWs in integrated IYCF-MNP interventions can also contribute to improved IYCF practices. In our study, the integration of MNP into the IYCF-MNP intervention may have contributed to both the supply and demand sides of IYCF counselling. On the supply side, the incorporation of evidence-based IYCF trainings [20], the social marketing performance-based incentive structure [26,27] and the introduction of a new tangible good for the CHWs to provide mothers in their community [26] may have improved CHW capacity and motivation for delivering IYCF messages. A recent systematic review of CHW performance found that when CHWs are able to provide curative services or physical goods, they feel more recognition from their community and ultimately more motivated [26]. Of note, the review included two studies from Madagascar: one showing that increased tasks (in particular having CHWs distribute injectable contraceptives) enhanced CHW motivation [28] and another Madagascar study showing that CHWs with a higher number of perceived responsibilities had better performance indicators than CHWs with low numbers of perceived responsibilities [29]. The Fortidom program may have also increased maternal demand for IYCF counselling—qualitative research by program staff highlighted several instances where mothers identified improvements in their children’s energy and mood after beginning MNP, which in turn encouraged them to continue MNP-use and attendance at CHW meetings. At the same time that Fortidom was distributing MNP through CHWs in Fénérive-Este and Vavatenina, in urban areas of Madagascar, Fortidom distributed MNP through facility-based health care providers. Although thorough baseline and endline surveys were not conducted in urban areas, programmatic data revealed lower rates of MNP purchases and adherence in urban areas indicating that the unique role of the CHW in the community may have contributed to higher rates of MNP purchases and consumption. Interviews with mothers in Vavatenina and Fénérive-Este during program roll-out revealed that the majority of mothers who tried MNP considered the CHW as their primary motivator, and that some mothers reported troubleshooting IYCF and MNP challenges with their CHW (such as the child refusing to eat MNP). 

In addition to Fortidom’s role in improving IYCF practices, we also found that the added micronutrients in MNP were likely an independent contributor to children’s hemoglobin level and risk of anemia. In models controlling for demographic characteristics, maternal CHW exposure and child’s dietary diversity, we found that children who consumed MNP had a significantly higher mean hemoglobin levels and a lower risk of anemia. A recent study which pooled dietary data from 15 studies in low-resource settings found that complementary feeding diets were consistently inadequate in several micronutrients, and that even when the researchers optimized children’s diets with modelling techniques, intake of several micronutrients remained inadequate [30]. In many settings—including in our Madagascar sample where dietary diversity remained below the recommended four food groups for the majority of children—interventions focusing on behavior change communication strategies promoting consumption of local complementary foods may not be sufficient to address the micronutrient gap. Of note, our integrated IYCF-MNP program was associated with increased intakes of vitamin A-rich fruits and vegetables and dairy among children aged 6–23 months, but we did not see changes in consumption of meat (which is particularly rich in micronutrients), likely due to barriers in cost and accessibility. Integrating MNP into IYCF interventions thus has the potential to help fill the micronutrient gap and ultimately decrease children’s risk of anemia and micronutrient deficiencies. 

Efficacy trials in low-resource settings have shown that MNP can reduce anemia prevalence by over 25% [7,8,31]. We found a smaller reduction in anemia, likely due to limited MNP coverage and adherence, which has been documented in other programmatic settings [12,13,14]. One major implementation barrier affecting Fortidom was a product quality problem that arose in the first few months of the program, which resulted in an unstable product that changed in consistency, color and taste. While the MNP remained safe for consumption and retained its nutritional value, the metallic taste discouraged children from eating foods with MNP. The program team spent three months recalling MNP from warehouses and field distribution sites in order to replace the supply with an improved product, and initiated monthly product quality checks at each of the supply centers; however, CHW stocks could not be recalled due to logistic constraints, and thus some mothers continued to use the altered product. Program staff encouraged CHWs to use revised behavior change messages acknowledging that MNP may change the texture, color and taste of the food slightly, but that this was normal (except in extreme scenarios, which were also explained). CHWs also conducted cooking demonstrations and counselled mothers on how to find locally-available foods that could help mask the taste of MNP (i.e., they encouraged mothers to mix MNP with foods with papaya, banana or yoghurt but not with plain rice). Of note, these revised messages may have contributed to the observed changes in dietary diversity. Despite these efforts, product quality issues may have contributed to the lack of trust among mothers of MNP. Ultimately, only half of mothers in our program area tried MNP, and among those who did, only half used ≥30 sachets. The most frequently cited reason that mothers reported not trying MNPs at endline was a lack of trust/understanding of MNP (36.6%), thus highlighting the importance of ensuring product quality, as well as maintaining a strong knowledge base, communication skills, and outreach activities of CHWs as vital to successful IYCF-MNP interventions. 

Despite the limited MNP coverage and adherence rates, the Fortidom program was still associated with a lower risk of anemia, a finding that is similar to a separate study assessing a social marketing distribution of MNP in Western Kenya [17]. The Kenya study found that although only 33% of households in the intervention area purchased MNP and average consumption in intervention villages was 0.9 sachets per week, the children in intervention villages still had a significantly higher mean hemoglobin and a lower risk of iron and vitamin A deficiencies than children in control villages. In both Fortidom and the Kenya program, stronger coverage and adherence likely would have resulted in greater improvements in micronutrient status; however, it is worth noting that programs using distribution strategies other than social marketing have also encountered barriers in coverage and adherence [12,13], and ultimately had smaller improvements in micronutrient status [14] compared to efficacy trials in controlled environments [7,32]. We did not find affordability to be a significant predictor of trying or continuing to use MNPs, and 94% of mothers who knew the price of MNP thought the product was affordable. A separate study from Niger found that mothers were willing to pay USD 0.03 per sachet of MNP [33], while the Kenya trial sold MNP for USD 0.027 to 33% of their target population [17]. Taken together, this evidence indicates that market-based approaches may be viable models for distributing MNP; however, none of the studies were specifically designed to assess the sale MNP compared to other distribution strategies, thus further research comparing distribution strategies for MNP is urgently needed.

Strengthening interventions to enhance consumption, particularly sustained consumption, has been identified as a key challenge facing MNP programs [34]; however, few studies have assessed which aspects of program implementation are associated with MNP coverage [12] and adherence [13]. We found that attending a CHW-IYCF talk and frequent exposure to IYCF-MNP radio messages were both associated with mothers’ initial use of MNP, but only CHW exposure was associated with continued MNP use. We also found that mothers who believed other mothers in the community were using MNP were more likely to try MNP, but only maternal confidence in her ability to explain the benefits of MNP was significantly associated with continued MNP use. Our results are similar to an evaluation of a pilot program in Nepal that found that mothers who attended a CHW-led mothers’ group meeting where MNP was discussed, and mothers who perceived positive effects of MNP in their children were significantly more likely to pick-up at least two batches of MNP [12]; they also found that mothers who perceived a positive effect of MNP were in turn more likely to feed their child at least 45 sachets [13]. The cross-sectional analyses in both programs limit our ability to infer whether maternal confidence (or maternal perception of positive benefits in Nepal) led to sustained MNP-use or vice versa; however, taken together, the evidence highlights the importance of a BCC intervention that emphasizes consistent CHW-exposure, shifting social norms, strong maternal understanding of MNP and IYCF practices, and a clear emphasis on the perceived benefits of MNP.

Limitations of our study include the cross-sectional nature of the baseline and endline surveys which were conducted in different seasons. It is possible some of the differences between baseline and endline in anemia risk are due to seasonal or secular trends. One important seasonal change that we are unable to fully account for in our study is seasonal changes in incidence of malaria, which in turn could affect the prevalence of anemia. We did not specifically assess malaria in our surveys, but interviewers were instructed to not assess hemoglobin in children who were currently ill, and we controlled our analyses for whether the child had been ill in the two weeks before the survey, and specifically whether the child had had a fever. Given that peak malaria season in our program areas is January–April (which includes our endline but not our baseline survey), and also that health facility data indicate that malaria incidence increased in Madagascar during the period of our program intervention, there may have been a higher prevalence of malaria among children in our endline survey than in our baseline survey. This in turn, would have contributed to higher rates of anemia at endline. Taken together, this indicates that were we able to fully account for the impact of malaria on anemia prevalence, we would have expected an even more robust effect of the Fortidom program on anemia. 

Another important limitation of our study is the potential role of seasonality and methodological differences in measurement of dietary diversity in baseline and endline. It is possible that seasonal differences in food availability contributed to the improved IYCF practices from baseline to endline. Of note, the two food groups with the largest changes from baseline to endline were vitamin-A-rich fruits and vegetables, and other fruits and vegetables. While we cannot fully account for changes in seasonal availability, several specific food items from both of these food groups were available, and consumed by children, in both baseline and endline seasons including papaya, sweet potatoes and wild greens for vitamin-A-rich fruits and vegetables; and bananas, jack fruit and tomatoes for other fruits and vegetables. Furthermore, our identification of significant differences in anemia and IYCF practices comparing MNP-users to non-users in the endline assessment, which occurred within a single season using uniform methodology, indicates that some of the improvements can likely be attributed to Project Fortidom. In analyses comparing MNP-users and non-users, we are further limited by the lack of randomization of exposure, thus underlying differences between mothers who tried MNP and mothers who did not may be confounding the association between program exposure and anemia and IYCF practices. We did, however, adjust for key demographic covariates, including maternal literacy and socioeconomic variables, which did not substantially change our findings. 

Our study has several strengths—it utilizes representative data from two rural districts where a large-scale, integrated IYCF and MNP intervention was implemented. We have ample baseline and endline sample sizes with thorough data on program exposure, children’s hemoglobin, and maternal IYCF knowledge and practices, as well as several sociodemographic confounders. In addition, our statistical analyses using log-binomial regression models directly estimate relative risks, as opposed to logistic regression models for odds ratios, which would have over-estimated the relative risks, particularly given the high prevalence of our outcomes [23,24].

## 5. Conclusions

Our study found that in the two districts in rural Madagascar where a community-based, integrated IYCF-MNP intervention was implemented, there was a significant reduction in the prevalence of anemia from baseline to endline, as well as significant improvements in maternal IYCF knowledge and practices, particularly relating to increased dietary diversity. In order to maximize the benefits of MNP interventions, future implementation research should focus on how to maximize MNP coverage and promote adherence. Given that attending a CHW-IYCF meeting and maternal confidence in her ability to explain the benefits of MNP were significant predictors of both trying MNP and consuming ≥30 sachets, future interventions should prioritize community-based distribution strategies that target maternal knowledge and confidence on MNP and IYCF in order to maximize the benefits of integrated IYCF-MNP interventions. 

## Figures and Tables

**Table 1:** Sociodemographic Characteristics of Study Sample ^1^.

	2012	2014
	*n* = 372 ^2^	*n* = 472 ^2^
**Household Characteristics**		
Urban	119 (32.1)	171 (36.2)
Household size		
1–3 people	83 (22.4)	158 (33.4)
4–5 people	169 (45.5)	198 (42.1)
≥6 people	119 (32.1)	116 (24.5)
Includes ≥2 children under 5 years	89 (24.0)	107 (22.7)
Main source of income		
Agriculture or livestock	270 (72.7)	291 (61.6)
Commercial Mining	28 (7.5)	73 (15.5)
Small business	31 (8.3)	30 (5.2)
Other	43 (11.5)	83 (17.7)
Household assets ^3^	-	-
0	151 (40.6)	172 (36.4)
1	147 (39.7)	161 (34.1)
2+	73 (19.7)	139 (29.5)
Ariary spent on food per person per day ^4^		
<500	120 (33.1)	80 (20.3)
500–1000	186 (51.5)	230 (58.7)
>1000	56 (15.4)	82 (21.80)
**Maternal Characteristics**		
Mother’s age	27.7 ± 8.9	27.1 ± 7.5
Maternal literacy ^5^		
able to easily read a newspaper	154 (41.9)	278 (59.0)
able to read newspaper with difficulty	100 (27.3)	124 (26.4)
unable to read a newspaper	113 (30.8)	69 (14.7)
**Child Characteristics**		
Male sex	186 (50.1)	224 (47.4)
Child’s age		
6–11 months	138 (37.1)	155 (32.8)
12–17 months	126 (33.9)	159 (33.6)
18–23 months	107 (28.9)	158 (33.6)

^1^ Frequency (%) for all categorical variables (may not add up to 100% due to rounding); Mean ± standard deviation for all continuous variables; ^2^ Analyses are weighted based on district population size; ^3^ From a three item list that includes: transportation (bicycle, car or truck), phone (cell phone or landline) and electronics (radio or television); ^4^ *n* = 362 in 2012 and 392 in 2014. In October 2012, 1USD was approx. 2200 ariary; in February 2014, 1USD was approx. 2300 ariary; ^5^ *n* = 368 in 2012 and 472 in 2014.

**Table 2:** Comparing maternal IYCF knowledge and practices, and children’s hemoglobin and anemia risk in households at endline versus baseline.

	2012 ^1^ *N* = 372	2014 ^1^ *N* = 475	Crude RR or Difference of Means (95% CI) ^2^	*p* Value	Adjusted RR or Difference in Means (95% CI) ^2,3^	*p* Value
**Maternal Nutrition Knowledge**						
Has heard of iron deficiency	52 (14.6)	177 (38.5)	2.63 (2.00, 3.47)	<0.001	2.41 (1.83, 3.18)	<0.001
Is able to identify a consequence of iron deficiency/anemia	46 (12.3)	334 (70.8)	5.77 (4.37, 7.62)	<0.001	5.48 (4.14, 7.25)	<0.001
Identifies children 0–23 months as particularly vulnerable to iron deficiency/anemia	14 (4.1)	175 (37.0)	9.12 (5.43, 15.32)	<0.001	8.48 (5.04, 14.26)	<0.001
**IYCF Practices in the previous day**						
Child was breastfed	305 (83.1)	388 (87.7)	1.05 (1.00, 1.12)	0.07	1.07 (1.01, 1.14)	0.02
Child received solid food (among children aged 6–8 months) ^4^	59 (83.9)	63 (84.7)	1.01 (0.88, 1.16)	0.90	0.99 (0.86, 1.14)	0.86
Child received solid food (among children aged 6–23 months)	346 (94.1)	451 (96.0)	1.02 (0.99, 1.05)	0.21	1.01 (0.97, 1.04)	0.71
Child consumed food from the following food groups in the previous day:						
Meat, fish or shellfish	141 (38.5)	212 (45.2)	1.17 (1.00, 1.38)	0.05	1.07 (0.91, 1.25)	0.42
Dairy	30 (8.1)	80 (17.1)	2.11 (1.42, 3.15)	<0.001	1.88 (1.27, 2.77)	0.002
Eggs	11 (2.9)	28 (6.0)	2.07 (1.04, 4.14)	0.04	1.68 (0.85, 3.31)	0.13
Legumes	102 (27.7)	127 (27.0)	0.98 (0.78, 1.22)	0.84	0.94 (0.75, 1.18)	0.57
Vitamin-A rich fruits and vegetables	177 (48.1)	355 (75.5)	1.57 (1.39, 1.76)	<0.001	1.52 (1.34, 1.73)	<0.001
Other fruits and vegetables	55 (15.1)	318 (67.6)	4.49 (3.49, 5.76)	<0.001	4.28 (3.30, 5.54)	<0.001
Grains, roots or tubers	345 (93.9)	453 (96.3)	1.03 (0.83, 1.17)	0.12	1.02 (0.98, 1.05)	0.35
Number of food groups consumed	2.35 ± 1.10	3.35 ± 1.31	1.00 (0.72, 1.07)	<0.001	0.86 (0.69, 1.02)	<0.001
Minimum dietary diversity (≥4 groups) ^5^	55 (14.9)	226 (48.0)	3.23 (2.48, 4.19)	<0.001	2.92 (2.24, 3.80)	<0.001
Frequency of solid/semi-solid food	3.08 ± 1.22	3.31 ± 1.35	0.23 (0.05, 0.41)	0.01	0.12 (−0.06, 0.29)	0.20
Minimum meal frequency ^5^	290 (79.7)	363 (77.8)	0.98 (0.91, 1.05)	0.50	0.95 (0.88, 1.03)	0.24
Minimum acceptable diet ^6^	49 (14.0)	177 (44.8)	3.20 (2.41, 4.24)	<0.001	2.88 (2.17, 3.82)	<0.001
Among sick children ^7^, child was fed more breastmilk than usual	39 (32.2)	108 (51.5)	1.60 (1.20, 2.14)	0.002	1.80 (1.32, 2.45)	<0.001
Among sick children ^7^, child was fed more solid food than usual	2 (1.6)	42 (20.0)	12.27 (2.99, 50.36)	<0.001	12.19 (2.96, 50.17)	<0.001
**Child’s hemoglobin and anemia risk**						
Mean hemoglobin (g/L) ^8^	102.4 ± 11.4	102.5 ± 14.1	0.1 (−1.7, 2.0)	0.88	0.5 (−1.4, 2.3)	0.64
Prevalence of anemia ^8^	247 (75.3)	281 (64.9)	0.86 (0.78, 0.95)	0.002	0.86 (0.78, 0.95)	0.003

^1^ Values are mean ± standard deviation for continuous variables and n (%) for categorical variables; values are weighted based on the population of each district. Where more than 5% of total sample is excluded from analysis, indicator-specific sample sizes are listed below; ^2^ Relative risks and differences of means compare endline households to baseline households (reference group). Relative risks and corresponding 95% confidence intervals and p-values were estimated using log-binomial regression models. When the log-binomial model did not converge, the poisson distribution was used. Difference of means and corresponding 95% confidence intervals and *p*-values were from linear regression models. Models were weighted based on the population of each district; ^3^ Multivariate models adjust for child’s sex and age (6–11, 12–17 or 18–23 months), district, urban vs. rural, household head’s occupation (agriculture/livestock or other), household asset score (0, 1 or ≥2 from 3-item list of transportation (bicycle or car), phone (cell or landline) and electronics (radio or television)), and maternal literacy (able to easily read a newspaper, able to read a newspaper with difficulty, or unable to read newspaper); ^4^ Sample of children aged 6–8 months: *n* = 70 in 2012; *n* = 74 in 2014; ^5^ Minimum meal frequency defined as ≥2× per day for breastfed infants aged 6–8 months, ≥3× for breastfed children aged 9–23 months, and ≥4× for non-breastfed children 6–23 months; ^6^ Minimum Acceptable Diet defined as minimum meal frequency and minimum dietary diversity in the previous 24 h for children who are breastfed, and defined as minimum meal frequency, minimum dietary diversity (4 out of 6 food groups excluding dairy), and at least two milk feedings for children who were not breastfed; ^7^ Sample size of children who have been sick in the previous 2 weeks: *n* = 121 in 2012 and *n* = 210 in 2014; ^8^ Sample sizes for hemoglobin and anemia were *n* = 328 in 2012; *n* = 433 in 2014.

**Table 3:** Comparing endline nutritional status and IYCF practices among users and non-users of MNPs.

	MNPs ^1^ *n* = 229	No MNPs ^1^ *n* = 245 (Reference)	Crude RR or Difference of Means (95% CI) ^2^	*p*	Adjusted RR or Difference of Means (95% CI) ^2,3^	*p*
**IYCF Practices**						
Child was breastfed yesterday	187 (86.2)	216 (91.1)	0.95 (0.88, 1.01)	0.10	1.02 (0.95, 1.09)	0.61
% of children 6–8 months who received solid/semi-solid food yesterday ^4^	19 (100.0)	54 (85.7)	cannot be estimated ^5^	-	cannot be estimated ^5^	-
% of children 6–23 months who received solid/semi-solid food yesterday	226 (98.7)	231 (94.3)	1.05 (1.01, 1.08)	0.01	1.03 (1.00, 1.07)	0.046
Number of food groups consumed yesterday	3.62 ± 1.15	3.14 ± 1.34	0.48 (0.25, 0.70)	<0.001	0.35 (0.13, 0.58)	0.002
Minimum dietary diversity (4+ food groups)	129 (56.3)	105 (42.9)	1.31 (1.09, 1.58)	0.004	1.22 (1.01, 1.47)	0.04
Frequency of solid/semi-solid food yesterday	3.46 ± 1.34	3.13 ± 1.38	0.33 (0.08, 0.57)	0.01	0.18 (−0.07, 0.43)	0.17
Received minimum meal frequency ^6^	177 (77.3)	186 (76.5)	1.01 (0.91, 1.11)	0.85	0.99 (0.90, 1.09)	0.84
Received minimum acceptable diet ^7^	104 (45.4)	95 (39.1)	1.16 (0.94, 1.44)	0.17	1.07 (0.86, 1.33)	0.54
Among sick children ^8^, child was fed more breastmilk than usual	47 (47.5)	52 (54.7)	0.87 (0.66, 1.14)	0.31	0.95 (0.72, 1.27)	0.74
Among sick children ^8^, child was fed more solid food than usual	20 (20.2)	24 (25.3)	0.80 (0.47, 1.35)	0.40	0.74 (0.43, 1.29)	0.29
**Hemoglobin level and anemia risk**						
Mean hemoglobin (g/L) ^9^	105.0 ± 13.7	100.6 ± 13.1	4.4 (1.9, 6.9)	<0.001	4.5 (1.9, 7.1)	<0.001
Prevalence of anemia ^9^	126 (59.2)	159 (70.7)	0.84 (0.73, 0.96)	0.01	0.86 (0.74, 0.99)	0.04

^1^ Values are mean ± standard deviation for continuous variables and n (%) for categorical variables. Where more than 5% of total sample is excluded from analysis, indicator-specific sample sizes are listed below; ^2^ Relative risks and corresponding 95% confidence intervals and *p*-values were estimated using log-binomial regression models. When the log-binomial model did not converge, log-poisson models were used. Differences of means and corresponding 95% confidence intervals and *p*-values are from linear regression models; ^3^ All multivariate models adjust for child’s sex and age (6–11, 12–17 or 18–23 months), household district and urban vs. rural, household head’s occupation (agriculture/livestock or other), household asset score (0, 1 or ≥2 from 3-item list of transportation (bicycle or car), phone (cell or landline) and electronics (radio or television)), and maternal literacy (able to easily read a newspaper, able to read a newspaper with difficulty, or unable to read newspaper). Models for hemoglobin and anemia also adjust for whether the mother attended a CHW talk on IYCF in the previous 3 months, the number of food groups the child consumed the previous day, and whether the child had been ill in the previous 2 weeks; ^4^ Sample size of children 6–8 months: *n* = 19 for MNP-users and *n* = 63 for non-users; ^5^ RR cannot be estimated because 100% of the 19 children 6–8 months who received MNPs received solid food yesterday; ^6^ Minimum meal frequency defined as solid or semi-solid foods ≥2× per day for breastfed infants aged 6–8 months, ≥3× for breastfed infants aged 9–23 months and ≥4× for non-breastfed infants aged 6–23 months; ^7^ Minimum acceptable diet defined as min. meal frequency and min. dietary diversity in the previous 24 h for children who are breastfed, and defined as minimum meal frequency, at least 4 out of 6 food groups (excludes dairy), and at least two milk feedings for children who were not breastfed; ^8^ Sample size for sick children: *n* = 99 for MNP-users and 95 for non-users; ^9^ Sample sizes for hemoglobin and anemia is *n* = 213 for MNP-users and *n* = 225 for non-users.

**Table 4:** Predictors of MNP Consumption.

	*N*	Number (%) MNP Users	Univariate		Multivariate ^2^	
			RR (95% CI) ^1^	*p*	RR (95% CI) ^1^	*p*
**Household Characteristics**						
District						
Fenerive-Est	200	104 (52.0%)	1.14 (0.95, 1.37)	0.17	0.91 (0.76, 1.10)	0.33
Vavatina	274	125 (45.6%)	reference	-	reference	-
Household Location						
Urban	214	84 (39.3)	reference	-	reference	-
Rural	260	145 (55.8)	1.42 (1.16, 1.73)	<0.001	1.43 (1.19, 1.74)	<0.001
Sex of household head						
Male	403	94 (48.1)	reference	-	-	-
Female	71	35 (49.3)	1.02 (0.79, 1.32)	0.86	-	-
Number of household members:						
1–3	148	86 (58.1)	1.28 (1.01, 1.62)	0.04	1.26 (1.00, 1.60)	0.05
4–5	205	88 (42.9)	0.94 (0.73, 1.21)	0.66	0.91 (0.72, 1.16)	0.46
≥6	121	55 (45.5)	reference	-	reference	-
Number of children under 5 years						
1	354	182 (51.4)	1.33 (1.04, 1.70)	0.02	0.99 (0.77, 1.27)	0.95
≥2	119	46 (38.7)	reference	-	reference	-
Main source of income						
Agriculture or livestock	313	160 (51.1)	reference	-	reference	-
Other	161	69 (42.9)	0.84 (0.68, 1.03)	0.10	0.85 (0.70, 1.05)	0.13
Household assets ^3^						
0	171	87 (50.9)	1.08 (0.85, 1.37)	0.53	-	-
1	180	84 (46.7)	0.99 (0.78, 1.26)	0.93	-	-
2+	123	58 (47.2)	reference	-	-	-
Ariary spent on food per person per day ^4^						
<500	103	51 (49.5)	1.05 (0.77, 1.43)	0.77	-	-
500–1000	232	111 (47.8)	1.01 (0.77, 1.34)	0.93	-	-
>1000	72	34 (47.2)	reference	-	-	-
**Maternal Characteristics**						
Mother’s Age						
youngest tertile (<23 years)	158	79 (50.0)	1.08 (0.86, 1.36)	0.48	-	-
middle tertile (23–29 years)	149	73 (49.0)	1.06 (0.84, 1.34)	0.61	-	-
oldest tertile (>29 years)	167	77 (46.1)	reference	-	-	-
Maternal Literacy						
able to easily read a newspaper	270	140 (51.9)	1.39 (1.03, 1.88)	0.03	1.36 (1.03, 1.80)	0.03
reads newspaper with difficulty	121	58 (47.9)	1.28 (0.92, 1.79)	0.14	1.17 (0.86, 1.60)	0.31
unable to read a newspaper	83	31 (37.4)	reference	-	reference	-
**Child Characteristics**						
Sex						
Male	233	116 (49.8)	reference	-	-	-
Female	241	113 (46.9)	0.94 (0.78, 1.13)	0.53	-	-
Child’s Age						
6–11 months	165	54 (32.7)	reference	-	reference	-
12–17 months	163	81 (49.7)	1.52 (1.16, 1.98)	0.002	1.51 (1.17, 1.94)	0.001
18–23 months	146	94 (64.4)	1.97 (1.53, 2.53)	<0.001	1.99 (1.57, 2.52)	<0.001
**Program Exposure**						
Mother attended a CHW meeting on IYCF in last 3 months						
Yes	105	70 (66.7)	1.55 (1.29, 1.85)	<0.001	1.48 (1.25, 1.77)	<0.001
No	369	159 (43.1)	reference	-	reference	-
In the past 3 months, mother heard IYCF radio messages:						
Everyday	182	111 (61.0)	1.97 (1.46, 2.65)	<0.001	1.96 (1.49, 2.59)	<0.001
At least once a week, but less than everyday	95	46 (48.4)	1.56 (1.11, 2.21)	0.01	1.65 (1.21, 2.25)	0.002
Less than once a week	84	37 (44.1)	1.42 (0.99, 2.05)	0.06	1.49 (1.06, 2.11)	0.02
Never	113	35 (31.0)	reference		reference	-
**Maternal Nutrition Knowledge**						
Mother has heard of iron deficiency						
Yes	192	94 (49.0)	1.00 (0.83, 1.20)	0.98	-	-
No	273	134 (49.1)	reference	-	-	-
Mother can name a consequence of anemia/iron deficiency						
Yes	342	167 (48.8)	1.04 (0.84, 1.28)	0.72	-	-
No	132	62 (47.0)	reference	-	-	-
Mother identifies children 0–23 months as particularly vulnerable to anemia/iron deficiency						
Yes	180	90 (50.0)	1.06 (0.87, 1.28)	0.56	-	-
No	294	139 (47.3)	reference	-	-	-
**Maternal Perceptions of MNP**						
Feels confident she can explain MNP benefits to others						
Yes	170	131 (77.1)	2.39 (1.99, 2.87)	<0.001	1.60 (1.37, 1.87)	<0.001
No	304	98 (32.2)	reference		reference	-
Identifies the following as primary location to get MNP						
CHW	293	194 (66.2)	1.49 (1.14, 1.95)	0.004	1.34 (1.06, 1.69)	0.01
Other source	72	32 (44.4)	reference	-	reference	-
Does not know location	109	3 (2.8)	0.06 (0.02, 0.19)	<0.001	0.08 (0.03, 0.25)	<0.001
Perceives other mothers in the community use MNP						
Yes	82	65 (79.3)	1.89 (1.61, 2.23)	<0.001	1.40 (1.22, 1.61)	<0.001
No	392	164 (41.8)	reference	-	reference	-
Believes her husband supports MNP						
Yes	377	184 (48.8)	1.02 (0.79, 1.31)	0.90	-	-
No	77	37 (48.1)	reference	-	-	-
Thinks that MNPs are affordable						
Yes	411	217 (52.8)	0.88 (0.61, 1.27)	0.50	-	-
No	20	12 (60.0)	reference	-	-	-

^1^ Relative risks, 95% confidence intervals and *p*-values were obtained from log-binomial regression models. When the log-binomial model would not converge, a log-poisson model was used; ^2^ Only variables that were significant at the *p* < 0.2 level in the univariate model were retained in the multivariate models. Maternal perception variables were excluded from primary multivariate models due to co-linearity with program exposure variables. Multivariate models witth maternal nutrition knowledge and perception of MNP variables were built separately; ^3^ From a three item list that includes: transportation (bicycle, car or truck), phone (cell phone or landline) and electronics (radio or television); ^4^ In February 2014, 1USD was approximately 2300 ariary.

**Table 5:** Predictors of consumption of ≥30 sachets of MNP among MNP users.

	*N*	Number (%) ≥30 Sachets	Univariate		Multivariate ^2^	
			RR (95% CI) ^1^	*p*	RR (95% CI) ^1^	*p*
**Household Characteristics**						
District						
Fenerive-Est	104	66 (63.5)	1.30 (1.03, 1.64)	0.03	1.32 (1.03, 1.69)	0.03
Vavatina	125	61 (48.8)	reference	-	reference	-
Household Location						
Urban	84	38 (45.2)	reference	-	reference	-
Rural	145	89 (61.4)	1.36 (1.04, 1.77)	0.03	1.12 (0.86, 1.46)	0.39
Sex of household head						
Male	194	106 (54.6)	reference	-	-	-
Female	35	21 (60.0)	1.10 (0.81, 1.48)	0.54	-	-
Number of household members						
1–3	86	53 (61.6)	1.41 (1.00, 1.99)	0.05	1.32 (0.93, 1.89)	0.12
4–5	88	50 (56.8)	1.30 (0.92, 1.85)	0.14	1.30 (0.92, 1.83)	0.14
≥6	55	24 (43.6)	reference	-	reference	-
Number of children under 5 years						
1	182	108 (59.3)	1.44 (1.00, 2.07)	0.05	1.35 (0.90, 2.03)	0.14
≥2	46	19 (41.3)	reference	-	reference	-
Main source of income						
Agriculture or livestock	160	90 (56.3)	reference	-	-	-
Other	69	37 (53.6)	0.95 (0.74, 1.23)	0.72	-	-
Household assets ^3^						
0	87	47 (54.0)	0.92 (0.69, 1.23)	0.58	-	-
1	84	46 (54.8)	0.93 (0.70, 1.25)	0.65	-	-
2+	58	34 (58.6)	reference	-	-	-
Ariary spent on food per person per day ^4^						
<500	51	29 (56.9)	1.38 (0.87, 2.20)	0.18	1.60 (1.01, 2.52)	0.04
500–1000	111	66 (59.5)	1.44 (0.94, 2.22)	0.09	1.38 (0.93, 2.05)	0.11
>1000	34	14 (41.2)	reference	-	reference	-
**Maternal Characteristics**						
Mother’s Age						
youngest tertile (<23 years)	79	44 (55.7)	1.05 (0.78, 1.39)	0.76	-	-
middle tertile (23–29 years)	73	42 (57.5)	1.08 (0.81, 1.44)	0.60	-	-
oldest tertile (>29 years)	77	41 (53.3)	reference	-	-	-
Maternal Literacy						
able to easily read a newspaper	140	73 (52.1)	1.08 (0.72, 1.60)	0.71	1.11 (0.73, 1.69)	0.62
able to read newspaper with difficulty	58	39 (67.2)	1.39 (0.93, 2.08)	0.11	1.06 (0.72, 1.57)	0.76
unable to read a newspaper	31	15 (48.4)	reference	-	reference	-
**Child Characteristics**						
Sex						
Male	116	58 (50.0)	reference	-	reference	-
Female	113	69 (61.1)	1.22 (0.97, 1.54)	0.09	1.25 (0.99, 1.58)	0.06
Child’s Age						
6–11 months	54	21 (38.9)	reference	-	reference	-
12–18 months	81	48 (59.3)	1.52 (1.04, 2.23)	0.03	1.43 (0.99, 2.08)	0.06
18–23 months	94	58 (61.7)	1.59 (1.10, 2.30)	0.01	1.61 (1.11, 2.33)	0.01
**Program Exposure**						
Mother attended CHW meeting on IYCF in last 3 months						
Yes	70	51 (72.9)	1.52 (1.23, 1.89)	0.001	1.58 (1.27, 1.98)	<0.001
No	159	76 (47.8)	reference	-	reference	-
In the past 3 months, mother heard IYCF radio messages:						
Everyday	111	46 (41.4)	1.14 (0.80, 1.63)	0.48	-	-
At least once a week, but less than everyday	46	25 (54.5)	1.06 (0.70, 1.60)	0.80	-	-
Less than once a week	37	19 (51.4)	1.00 (0.64, 1.57)	0.99	-	-
Never	35	18 (51.4)	reference	-	-	-
**Maternal Nutrition Knowledge**						
Mother has heard of iron deficiency						
Yes	94	43 (45.7)	0.73 (0.56, 0.94)	0.02	1.20 (0.92, 1.57)	0.18
No	134	84 (62.7)	reference	-	reference	-
Mother can name a consequence of anemia/iron deficiency						
Yes	167	88 (52.7)	0.84 (0.66, 1.06)	0.15	0.98 (0.76, 1.26)	0.88
No	62	39 (62.9)	reference	-		
Mother identifies children 0–23 months as particularly vulnerable to anemia/iron deficiency						
Yes	90	51 (56.7)	1.04 (0.82, 1.31)	0.77	-	-
No	139	76 (54.7)	reference	-	-	-
**Maternal Perceptions of MNP**						
Feels confident she can explain MNP benefits to others						
Yes	131	84 (64.1)	1.46 (1.13, 1.89)	0.004	1.37 (1.02, 1.83)	0.03
No	98	43 (43.9)	reference	-	reference	-
Identifies the following as primary location to get MNP						
CHW	194	108 (55.7)	0.94 (0.69, 1.28)	0.69	-	-
Other source	32	19 (59.4)	reference	-	-	-
Does not know where to get MNP	3	0 (0.0)	-	-		
Perceives other mothers in the community use MNP						
Yes	65	38 (58.5)	1.08 (0.84, 1.38)	0.56	-	-
No	164	89 (54.3)			-	-
Believes her husband supports MNP						
Yes	184	23 (62.2)	0.88 (0.67, 1.17)	0.39	-	-
No	37	101 (54.9)			-	-
Thinks that MNPs are affordable						
Yes	217	122 (56.2)	1.35 (0.68, 2.66)	0.39	-	-
No	12	5 (41.7)	reference	-	-	-

^1^ Relative risks, 95% confidence intervals and p-values were obtained from log-binomial regression models. When the log-binomial model did not converge, a log-poisson model was used; ^2^ Only variagbles that were significant at the *p* < 0.2 level in the univariate models were retained in the multivariate models. Maternal perception variables were excluded from primary multivariate models due to co-linearity with program exposure variables. Multivariate models for maternal perception of MNP were built separately; ^3^ From a three item list that includes: transportation (bicycle, car or truck), phone (cell phone or landline) and electronics (radio or television); ^4^ In February 2014, 1USD was approximately 2300 ariary.
